# Endocrine Disruptors and Polycystic Ovary Syndrome: Phthalates

**DOI:** 10.4274/jcrpe.galenos.2020.2020.0037

**Published:** 2020-11-25

**Authors:** Leyla Akın, Mustafa Kendirci, Figen Narin, Selim Kurtoglu, Nihal Hatipoglu, Ferhan Elmalı

**Affiliations:** 1 Erciyes University Faculty of Medicine, Department of Pediatric Endocrinology, Kayseri, Turkey; 2 Ondokuz Mayıs University Faculty of Medicine, Department of Pediatric Endocrinology, Samsun, Turkey; 3 Erciyes University Faculty of Medicine, Department of Biochemistry, Kayseri; İzmir Kâtip Çelebi University, Department of Medical Biochemistry, İzmir, Turkey

**Keywords:** Phthalate, di-(2-ethylhexyl)-phthalate, mono-(2-ethylhexyl)-phthalate, endocrine disrupter, polycystic ovary syndrome

## Abstract

**Objective:**

We aimed to investigate a possible role of the endocrine disruptors phthalates, di-2-ethylhexyl phthalate (DEHP) and mono (*2-ethylhexyl*) phthalate (MEHP), in polycystic ovary syndrome (PCOS) aetiopathogenesis. We also wished to evaluate the relationship between phthalates and metabolic disturbances in adolescents with PCOS.

**Methods:**

A total of 124 adolescents were included. Serum MEHP and DEHP levels were determined by high-performance liquid chromatography. Insulin resistance was evaluated using homeostasis model assessment-insulin resistance, quantitative Insulin Sensitivity Check Index, fasting glucose/insulin ratio, Matsuda index, and total insulin levels during oral glucose tolerance test. Participants were further subdivided into lean and obese subgroups according to body mass index (BMI).

**Results:**

Sixty-three PCOS and 61 controls, (mean age 15.2±1.5; range: 13-19 years) were enrolled. Serum DEHP and MEHP concentrations were not significantly different between PCOS and control groups. The mean (95% confidence interval) values of DEHP and MEHP were 2.62 (2.50-2.75) μg/mL vs 2.71 (2.52-2.90) μg/mL and 0.23 (0.19-0.29) μg/mL vs 0.36 (0.18-0.54) μg/mL in PCOS and the control groups respectively, p>0.05. Correlation analysis, adjusted for BMI, showed that both phthalates significantly correlated with insulin resistance indices and serum triglycerides in adolescents with PCOS.

**Conclusion:**

Serum DEHP and MEHP concentrations were not different between adolescents with or without PCOS. However, these phthalates are associated with metabolic disturbances such as dyslipidemia and insulin resistance, independently of obesity, in girls with PCOS.

**What is already known on this topic?**

In animal and in vitro studies, there are some significant findings suggesting that di-2-ethylhexyl phthalate (DEHP) and/or mono (2-ethylhexyl) phthalate (MEHP) might have a role in the development of polycystic ovary syndrome (PCOS). There are only a few human studies exploring the relationship between PCOS and phthalates in the literature.

**What this study adds?**

We found significant correlations between DEHP/MEHP and insulin resistance in adolescents with PCOS, suggesting that phthalates might have a possible effect on energy metabolism in this population.

## Introduction

Phthalates are a group of industrial chemicals that are commonly used as plasticisers in the production of soft toys, flooring, food packaging, paints, plastic bags, medical devices, cosmetics, and air fresheners (1). Di-2-ethylhexyl phthalate (DEHP), the most commonly used phthalate, and its metabolites including mono (2-ethylhexyl) phthalate (MEHP), are known to be endocrine disruptors and have been related to some health problems such as obesity (2), abnormal genital development (3), low semen quality (4), precocious puberty (5), and gynaecomastia (6) in humans.

Polycystic ovary syndrome (PCOS) is a common endocrine disorder characterised by menstrual irregularity, hyperandrogenism (HA) and polycystic ovaries. The pathogenesis of the disorder has not yet been fully clarified. In several animal studies, DEHP exposure has been shown to result in prolonged oestrous cycles and decreased ovulation rate (7,8). Davis et al (8) demonstrated that DEHP exposure caused hypo-oestrogenic, hypo-progestinic anovulatory cycles in previously regularly menstruating rats. Morphologically, polycystic ovaries developed in these rats.

There are only a few human studies investigating the relationship between PCOS and phthalates in the literature. However, the reported findings are not in agreement (9,10,11,12).

The aim of the present study was to evaluate serum MEHP and DEHP concentrations in adolescents with PCOS and compare with healthy controls. In addition, we wished to investigate the possible relationship between these endocrine disruptors and metabolic abnormalities in this population.

## Methods

The study was approved by the Ethical Committee of Erciyes University (approval no: 211-159). An informed consent was taken from each adolescent in addition to an informed consent obtained from the parents of the participants.

The study included adolescent girls who presented to our Paediatric Endocrinology Outpatient Clinic because of irregular menstrual bleeding and/or hirsutism between January 2011 and August 2012. An age-matched cohort who had regular menstruation and did not have hirsutism served as the control group. Exclusion criteria included the presence of any major disease, a history of taking insulin sensitising or antiandrogenic medication within the past one year and the current use of any drugs.

The participants partly served as the study population of another study, which has been published previously in which the methods of anthropometric measurements, assays, and the definitions of obesity, PCOS and insulin resistance are given in detail (13). In brief, modified Rotterdam criteria (14) was used for the diagnosis of PCOS and the adolescents who had a body mass index (BMI) ≥95^th^ percentile according to age and sex were defined as obese (15). Subjects required any two of the following three criteria to be present to be diagnosed with PCOS: oligo/anovulation (OA), clinical and/or biochemical evidence of HA and polycystic ovarian morphology (PCOM) on ultrasound, with other endocrinopathies excluded. At the beginning of the study 112 adolescents were diagnosed with PCOS. Twenty-six of the patients had classic phenotype (phenotype 1) with HA, OA and PCOM. Thirty-seven patients had only OA and HA (phenotype 2), 46 patients had only HA and PCOM (phenotype 3) and three patients had only OA and PCOM (phenotype 4). However, as the current Endocrine Society guidelines (16,17) recommend the presence of both HA and chronic anovulation for PCOS diagnosis in adolescence, we excluded the patients with phenotypes 3 and 4. Therefore, in total 63 girls with PCOS were included in the study. All the participants were at least 1-year postmenarcheal at their inclusion into the study. The presence of symptoms of oligomenorrhoea for at least two years was required to be used as a criterion for PCOS diagnosis. Biochemical HA was defined as serum total testosterone concentration ≥55 ng/dL. Hirsutism was diagnosed when Ferriman-Gallwey score was eight or more. When 17-hydroxyprogesterone (17-OH-P) concentrations were above 2 ng/mL, an adrenocorticotropic hormone (ACTH) stimulation test was carried out for differential diagnosis of congenital adrenal hyperplasia. Homeostasis model assessment-insulin resistance (HOMA-IR), Quantitative Insulin Sensitivity Check Index (QUICK-I), fasting glucose/fasting insulin ratio (FGIR), Matsuda index and total insulin levels during oral glucose tolerance test were used for the estimation of insulin resistance and sensitivity indexes (18).

Venous blood samples, for DEHP and MEHP measurement, were taken into glass test tubes to avoid plastic contamination. A clean aluminium foil was used to cover the mouths and surrounds of the tubes to protect the sample from contact with the screw caps and sunlight. The serum was separated by centrifugation at 800 g, and the samples were immediately taken into glass vials to be stored in a freezer at -80 °C until analysis.

The blood samples for the hormonal assays, including serum 17-OH-P, androstenedione, dehydroepiandrosterone sulphate (DHEA-S), total and free testosterone, follicle stimulating hormone (FSH), luteinising hormone (LH), oestradiol, and sex hormone binding globulin (SHBG) were taken in the morning during the early follicular phase (second to fifth day) of a spontaneous menstrual cycle or at any time in patients with amenorrhoea. Progesterone was measured in the second phase of the menstrual period. A chemiluminescence immunoassay method (Siemens Healthcare Diagnostics Products, Llanberis, UK) was used to measure serum prolactin, thyrotropin (TSH), free thyroxine free triiodothyronine, LH, FSH, and oestradiol concentrations. A further chemiluminescence immunoassay method (Siemens Healthcare Diagnostics Inc., Flanders, USA) was used to test plasma cortisol, and serum ACTH and insulin concentrations. Serum concentrations of 17-OH-P, DHEA-S, androstenedione, total testosterone and free testosterone were measured by a radioimmunoassay method. SHBG concentrations were analysed by immunoradiometric assay.

Routine enzymatic methods on an Abbott Architect c16000 analyser (Abbott Diagnostics, USA) were used to test serum fasting glucose, triglycerides, high-density lipoprotein-cholesterol (HDL-C), total cholesterol, alanine aminotransferase (ALT) and aspartate aminotransferase (AST) concentrations. An oral glucose tolerance test (OGTT) was performed using a dose of 1.75 g glucose/kg body weight (maximum 75 g) in all PCOS patients and obese controls. Venous blood samples were obtained at 0, 30, 60, 90, and 120 minutes to measure plasma glucose and plasma insulin concentrations in the morning, after an overnight fast.

### DEHP and MEHP Measurements

Determination of MEHP and DEHP concentrations was performed on a high-performance liquid chromatography (HPLC) analyzer equipped with an auto-sampler (Hewlett Packard Agilent 1100 Series, Vienna, Austria) and using a UV detector (230 nm). A Spherisorb C18 ODS2 column was used (250 mm x 4.6 mm I.D., 5 µm, Waters, Milford, MA, USA). Separations were performed at room temperature. The mobile phase was orthophosphoric acid 0.1% (acetonitrile [90:10, vol/vol]), and the flow rate was 1 mL/min. For the extractions, to a sample of 200 µL serum were added 400 µL of Na OH 1N, 100 µL of 50% H3PO4 and 600 µL of acetonitrile. After each addition, the sample was agitated by vortex for 30 seconds. After centrifugation for 10 minutes at 3500 rpm, the supernatant was separated, and the residue was again extracted with 600 µL of acetonitrile. After centrifugation under the same conditions, the collected supernatants were evaporated, reconstituted with 400 µL of mobile phase and injected into the chromatograph. The injection volume was 100 µL. Stock solutions containing DEHP or MEHP (2000 ppm) were prepared by dissolving a weighed amount of substance in acetonitrile. Standard solutions were prepared by dilution of the above stock solutions with the mobile phase and by varying the concentration in the range 0.05-5.0 ppm (6,19). The concentrations of DEHP and MEHP in the samples were calculated by using the calibration curve of the peak area prepared for DEHP and MEHP standards. The detection limits were determined as 0.05 ppm for DEHP and as 1 ppm for MEHP.

The retention times for DEHP and MEHP were 23 minutes and 3.7 minutes, respectively. Recovery studies were performed on blank samples of serum, and the mean±SD percentage recoveries were found to be 92±1.12% for DEHP and 99±1.10% for MEHP on 20 occasions. Between-run coefficient of variation (CV) were 6.44±0.12% for DEHP and 8.03±1.05% for MEHP. Within-day precisions were 8.75±0.43% CV for DEHP and 4.83±0.21% for MEHP. DEHP and MEHP were purchased from Merck (Merck GmbH, Hohenbrunn, Germany) and Cambridge Isotope Laboratories (Cambridge Isotope Laboratories Inc., Andover, MA, USA), respectively. Acetonitrile (HPLC grade) and all other analytical-grade reagents were obtained from Sigma-Aldrich Co. (Sigma-Aldrich Co., St Louis, MO, USA).

### Statistical Analysis

The IBM SPSS Statistics, version 21.0, statistics program was used to perform all statistical analyses (IBM Inc., Armonk, NY, USA). Data are given as frequencies or means with 95% confidence intervals. The distributions of continuous variables were analysed in terms of skewness and kurtosis and were transformed logarithmically, when appropriate. We used t-test to test differences between the groups. Mann-Whitney U was used for variables without normal distributions. The comparison of prevalences was performed by using chi-square test. We used correlation tests (Pearson or Spearman as appropriate) to analyse the relationship between the parameters. The value of p<0.05 was taken for statistical significance.

## Results

Subjects with PCOS consisted of 63 adolescent girls; 36 obese and 27 lean with a mean age of 15.3±1.3 (range: 13-19 years). The control group consisted of 61 age-matched healthy female adolescents (35 obese and 26 lean). The clinical and laboratory characteristics of the study population are shown in Tables 1 and 2. Serum DEHP and MEHP concentrations were not different between adolescent girls with or without PCOS (Table 2). In bivariate correlation analysis, correlations were investigated between the phthalates, DEHP and MEHP, and the following parameters: age, FSH, LH, oestradiol, progesterone, total and free testosterone, androstenedione, 17-OH-P, DHEA-S, BMI, waist circumference, glucose, triglyceride, HDL, low-density lipoprotein, total cholesterol, ALT, AST, insulin, HOMA-IR, QUICKI, Matsuda index, FGIR, and total insulin levels during OGTT. Serum MEHP and DEHP levels were correlated (r=0.32, p=0.02). We did not find any correlations between DEHP or MEHP and androgens, sex steroids and gonadotropins in either the entire group or in the PCOS subgroup. In contrast, significant correlations were present between MEHP or DEHP and insulin resistance indices, as well as serum lipids, in patients with PCOS. The parameters with statistically significant correlations with MEHP or DEHP in the PCOS group are shown in Table 3. These correlations were even more pronounced in the obese PCOS subgroup (Table 4). To eliminate the possible effect of obesity, we performed correlation analysis after adjustment for BMI in adolescents with PCOS, which revealed that both phthalates remained significantly correlated with insulin resistance indices and serum triglycerides (Table 5). There was no correlation between DEHP or MEHP and any other parameters, neither in the control group nor in the obese non-PCOS subgroup, when taken separately.

## Discussion

Although the evidence is limited, accumulating data are indicating the potential role of endocrine disruptors in the pathogenesis of adipogenesis and diabetes (20). It has been reported that DEHP exposure and insulin resistance are associated in adolescents (21). Insulin resistance, which is a well-known aetiological factor in PCOS development, is reported in 50-80% of women with PCOS (22,23). To our knowledge, this is the first report of an association of phthalates with insulin resistance in a PCOS cohort in the English literature.

In the current study, we found significant correlations between DEHP and insulin resistance and dyslipidaemia, suggesting that DEHP might have a direct or indirect effect on energy metabolism. This association was even stronger in the PCOS group and absent in the control group. Interestingly, in the PCOS group, after adjustment for BMI, the correlations of both DEHP and MEHP with insulin resistance indices and serum triglycerides remained strikingly significant. These findings lead us to think that there might be another mechanism, other than obesity, by which phthalates affect insulin resistance-one of the key pathologies in PCOS development - and dyslipidaemia in patients with PCOS.

In animal and *in vitro* studies, there are some significant findings suggesting that phthalates might have a role in the aetiopathogenesis of PCOS (7,8,24,25,26,27,28,29,30). Previous studies showed that DEHP exposure in rats results in prolonged oestrous cycles and decreased ovulation rates, altered circulating FSH, LH, testosterone, and progesterone levels (7,8,24,25). DEHP exposure resulted in suppressed oestradiol levels in granulosa cells which could not stimulate the LH surge necessary for ovulation. This consequently caused hypo-estrogenic anovulatory cycles and polycystic ovaries in adult female rats (8). Moreover, MEHP stimulates basal steroidogenesis (25), inhibits progesterone production in rat granulosa cells and decreases aromatase concentrations causing a hyperandrogenaemic, hypo-progestinic milieu which is similar to that seen in PCOS (29). In humans, in a study on granulosa-lutein cells from women planning *in vitro* fertilisation, MEHP was reported to inhibit oestradiol production and affected steroidogenesis similarly to the findings in rats (31).

Based on the results of these studies and given the fact that hyperfunctioning of theca and relative hypofunctioning of granulosa cells accompany the acyclicity of PCOS in humans, we thought that it was worthwhile investigating the PCOS-phthalate relationship in humans. In another paper inspiring us to conduct this study, Svechnikova et al (32) reported that DEHP exposure in female rats caused increased LH response to GnRH by pituitary and inhibition of progesterone production in granulosa cells.

There are only four studies in the literature, investigating PCOS-phthalate relationship in humans, which report conflicting findings (9,10,11,12). In the present study, we did not find any relation between DEHP or MEHP and gonadotropins or sex hormones. It is known that the effects of phthalates on hormones are both complex and multifactorial. Hence, considering the results of experimental studies, one might suggest that random measurements of serum concentrations of these compounds are not enough to elucidate a causal relationship between DEHP/MEHP and the disorder. Phthalates have been reported to be associated with gynecomastia and the risk of shortened anogenital distance (6,33). It may be argued that higher levels of DEHP/MEHP might mask PCOS findings, due to its antiandrogenic effects, which could be investigated in a non-hyperandrogenic PCOS group, such as in patients with Rotterdam criteria phenotype 4 (14).

In a very recent study, Jin et al (10) reported significantly increased DEHP levels in follicular fluid of women with PCOS compared to controls. They also showed that DEHP is associated with lower pregnancy rate and DEHP exposure resulted in significant increase in androgen production in human granulosa cells. We speculate that, in our cohort, DEHP might have a role in PCOS development through insulin resistance at the follicular level, which is not reflected in the serum concentrations of the participants.

### Study Limitations

One of the limitations of this study is its cross-sectional design, which does not allow for the identification of any causal relationship. The second limitation was that we measured serum FSH, LH, progesterone, and phthalate levels at different times of the menstrual cycle in patients, which might have also masked some associations.

## Conclusion

In this study, serum DEHP and MEHP concentrations in adolescents with PCOS were not different from those in their non-PCOS peers. However, both DEHP and MEHP significantly correlated with insulin resistance and metabolic disturbances in patients with PCOS. We believe that further well-designed studies are needed to evaluate the possible role of phthalates in PCOS development in humans.

## Figures and Tables

**Table 1:**
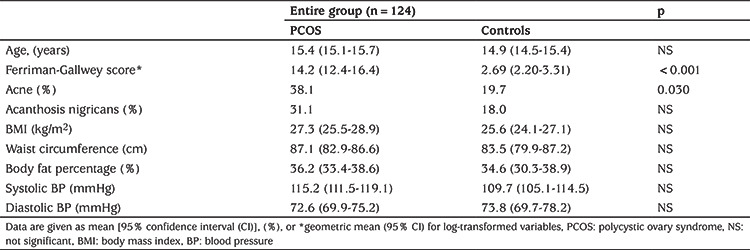
Clinical features of adolescents with and without polycystic ovary syndrome

**Table 2:**
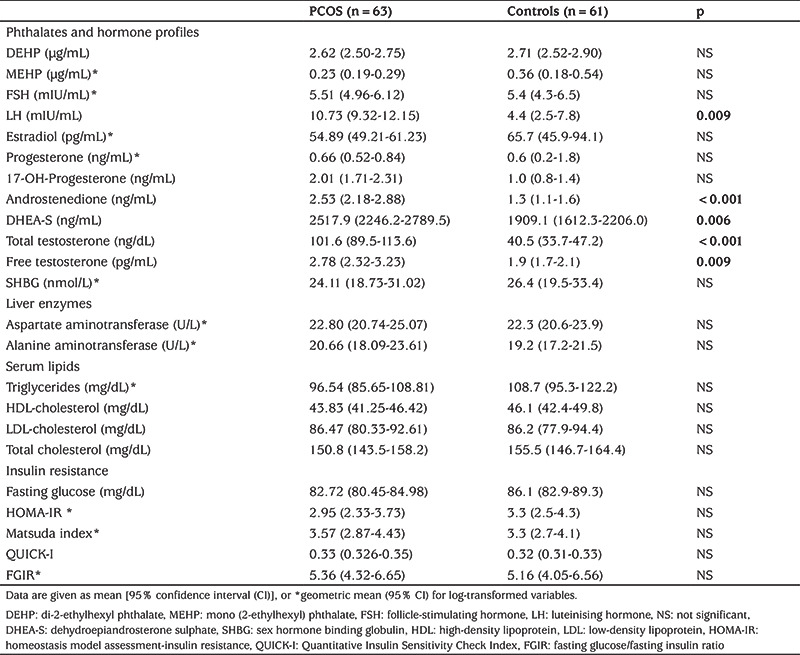
Laboratory measurements and serum di-2-ethylhexyl phthalate and mono (2-ethylhexyl) phthalate concentrations in adolescents with and without polycystic ovary syndrome

**Table 3:**
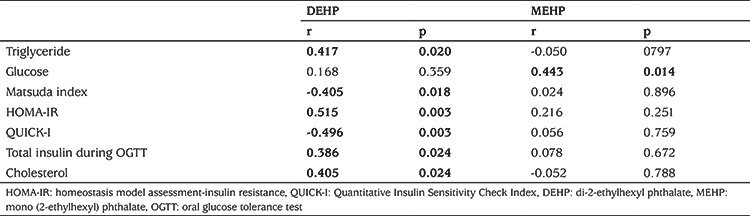
Correlations between mono (2-ethylhexyl) phthalate or di-2-ethylhexyl phthalate and metabolic parameters in adolescents with polycystic ovary syndrome

**Table 4:**
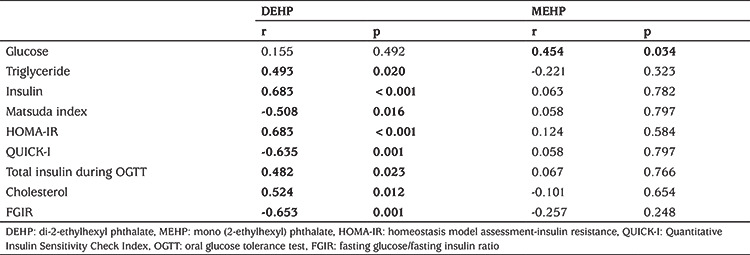
Correlations between mono (2-ethylhexyl) phthalate or di-2-ethylhexyl phthalate and metabolic parameters in obese adolescents with polycystic ovary syndrome

**Table 5:**
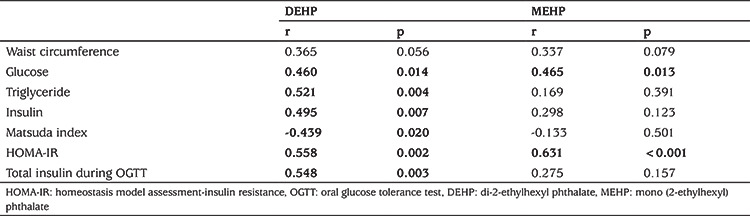
Correlations of mono (2-ethylhexyl) phthalate and di-2-ethylhexyl phthalate with metabolic parameters adjusted for body mass index in adolescents with polycystic ovary syndrome
